# Cocktail of Ropivacaine, Morphine, and Diprospan Reduces Pain and Prolongs Analgesic Effects after Total Knee Arthroplasty: A Prospective Randomized Controlled Trial

**DOI:** 10.1155/2024/3697846

**Published:** 2024-02-28

**Authors:** Zhenyu Luo, Weinan Zeng, Xi Chen, Qiang Xiao, Anjing Chen, Jiali Chen, Haoyang Wang, Zongke Zhou

**Affiliations:** ^1^Department of Orthopedics, Institute of Orthopedic Research, West China Hospital, West China School of Medicine, Sichuan University, Chengdu 610041, China; ^2^Department of Operative Dentistry and Endodontics, West China Hospital of Stomatology, Sichuan University, Chengdu 610041, China; ^3^Department of Orthopedics, West China Hospital, West China School of Nursing, Sichuan University, Chengdu 610041, China

## Abstract

**Background:**

Local infiltration analgesia (LIA) provides postoperative analgesia for total knee arthroplasty (TKA). The purpose of this study was to evaluate the analgesic effect of a cocktail of ropivacaine, morphine, and Diprospan for TKA.

**Methods:**

A total of 100 patients from September 2018 to February 2019 were randomized into 2 groups. Group A (control group, 50 patients) received LIA of ropivacaine alone (80 ml, 0.25% ropivacaine). Group B (LIA group, 50 patients) received an LIA cocktail of ropivacaine, morphine, and Diprospan (80 ml, 0.25% ropivacaine, 0.125 mg/ml morphine, and 62.5 *μ*g/ml compound betamethasone). The primary outcomes were the levels of inflammatory markers C-reactive protein (CRP) and interleukin-6 (IL-6), pain visual analog scale (VAS) scores, opioid consumption, range of motion (ROM), functional tests, and sleeping quality. The secondary outcomes were adverse events, satisfaction rates, HSS scores, and SF-12 scores. The longest follow-up was 2 years.

**Results:**

The two groups showed no differences in terms of characteristics (*P*  >  0.05). Group B had lower resting VAS pain scores (1.54 ± 0.60, 95% CI = 1.37 to 1.70 vs. 2.00 ± 0.63, 95% CI = 2.05 to 2.34) and active VAS pain scores (2.64 ± 0.62, 95% CI = 2.46 to 2.81 vs. 3.16 ± 0.75, 95% CI = 2.95 to 3.36) within 48 h postoperatively than Group A (*P* < 0.001), while none of the pain differences exceeded the minimal clinically important difference (MCID). Group B had significantly lower CRP levels (59.49 ± 13.01, 95% CI = 55.88 to 63.09 vs. 65.95 ± 14.41, 95% CI = 61.95 to 69.94) and IL-6 levels (44.11 ± 13.67, 95% CI = 40.32 to 47.89 vs. 60.72 ± 15.49, 95% CI = 56.42 to 65.01), lower opioid consumption (7.60 ± 11.10, 95% CI = 4.52 to 10.67 vs. 13.80 ± 14.68, 95% CI = 9.73 to 17.86), better ROM (110.20 ± 10.46, 95% CI = 107.30 to 113.09 vs. 105.30 ± 10.02, 95% CI = 102.52 to 108.07), better sleep quality (3.40 ± 1.03, 95% CI = 3.11 to 3.68 vs. 4.20 ± 1.06, 95% CI = 3.90 to 4.49), and higher satisfaction rates than Group A within 48 h postoperatively (*P*  <  0.05). Adverse events, HSS scores, and SF-12 scores were not significantly different within 2 years postoperatively.

**Conclusions:**

A cocktail of ropivacaine, morphine, and Diprospan prolongs the analgesic effect up to 48 h postoperatively. Although the small statistical benefit may not result in MCID, the LIA cocktail still reduces opioid consumption, results in better sleeping quality and faster rehabilitation, and does not increase adverse events. Therefore, cocktails of ropivacaine, morphine, and Diprospan have good application value for pain control in TKA. This trial is registered with ChiCTR1800018372.

## 1. Introduction

Total knee arthroplasty (TKA) is an effective surgical operation for the treatment of end-stage knee arthritis that may relieve knee pain and improve knee function and quality of life [1]. However, 19% of recipients are not satisfied with their operations [2]. The surgical trauma leads to inflammation that will aggravate the postoperative pain of the knee, cause swelling around the knee, and delay the recovery of joint function. Knee pain after TKA is the main reason why some patients are afraid of the operation, and knee pain affects the patient satisfaction rate. Therefore, it is necessary to formulate countermeasures to reduce postoperative knee pain and inhibit inflammatory reactions in TKA.

Pain management is an important part of the concept of enhanced recovery after surgery (ERAS) [3]. Multimodal analgesia is an effective combination of different analgesics and analgesic methods and is of great significance in controlling perioperative pain and reducing the use of postoperative opioids and related adverse reactions [4, 5]. Local injection analgesia (LIA) refers to the method of injecting mixed drugs into all layers of the joint tissue [6], also known as “cocktail analgesia.” LIA can directly reach the pain site, eliminate pain at the source, and preserve muscle strength, all of which are conducive to postoperative functional exercise and recovery and reduce the use of opioids after the operation [7]. However, there is no clear uniform standard for dispensing drugs.

Ropivacaine is the main component of LIA. However, its anesthetic effect is weak, and its duration is relatively short. It is usually necessary to combine it with other drugs to enhance the anesthetic effect and prolong the analgesic time. Morphine is an opioid drug of central analgesia, and local injection can allow morphine to be slowly absorbed, increase its action period, and reduce the central side effects of systemic administration [8]. However, the effects and duration of analgesia after adding morphine alone to LIA remain controversial. Some studies have shown that morphine has a short analgesic period and may increase the risk of postoperative nausea and vomiting (PONV) [9, 10]. Glucocorticoids reduce inflammation around the knee and significantly reduce pain and improve function [11–14]. They can also inhibit central nausea and vomiting [15, 16]. Diprospan is a long-acting glucocorticoid containing betamethasone sodium phosphate and betamethasone dipropionate, which can slow the absorption of betamethasone and relieve symptoms for an extended period. However, the effect and safety of adding morphine and betamethasone to LIA remain controversial.

The aim of this randomized controlled trial was to evaluate the efficacy and safety of adding morphine and betamethasone to cocktail therapy after TKA and to investigate whether the addition of morphine and betamethasone can increase the anesthetic effect and prolong the analgesic effect. We hypothesized that the addition of morphine and Diprospan may significantly reduce the inflammatory reaction, prolong the analgesic effect, improve pain relief, and not increase adverse events.

## 2. Materials and Methods

This prospective blinded randomized controlled trial has been reported in line with the Consolidated Standards of Reporting Trials (CONSORT) Guidelines. The trial was previously approved by the Clinical Trials and Biomedical Ethics Committee (2012268) and was also registered in the Clinical Trial Registry (ChiCTR1800018372). Informed consent was obtained from all patients.

**Figure 1:**
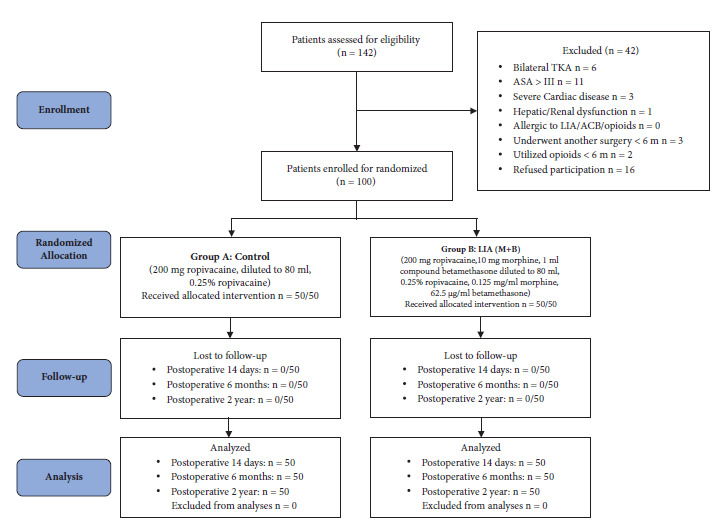
Study flowchart.

Patients who were over 18 years old, underwent primary unilateral TKA, and had an American Society of Anesthesiologists (ASA) physical health classification [17] less than Grade III were enrolled between September 2018 and February 2019. The exclusion criteria were patients declining participation; active local or systemic infection; allergies to local analgesia, opioids, or corticosteroids; use of opioids or corticosteroids within 6 months; severe liver or renal dysfunction; cardiac comorbidities; and pregnancy. Finally, 100 patients were included and randomized into 2 groups using a computer-generated list of random numbers. The allocation procedure is shown in Figure 1. Each patient was randomly assigned a random number; odd-numbered patients were assigned to Group A, and even-numbered patients were assigned to Group B. Grouping and dispensing were carried out by specialized nurses. Patients and the researchers performing TKA (supervised by the corresponding author) were blinded, and the study group was revealed at the last follow-up period.

**Figure 2:**
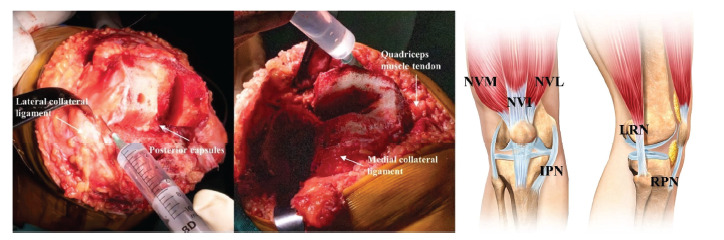
LIA technique diagram. The LIA was injected into the following layers: around the medial and lateral collateral ligament; the medial, lateral, and posterior capsules; the vastus medialis obliquus muscle and quadriceps tendon; and the prepatellar tissues and subcutaneous tissues. These site injections caused the following nerve endings that innervate the knee to be blocked. NVI, nerve to vastus intermedius; NVL, nerve to vastus lateralis; NVM, nerve to vastus medialis; LRN, lateral retinacular nerve from sciatic nerve; IPN, infrapatellar branch of saphenous nerve; RPN, recurrent peroneal nerve from common peroneal nerve.

Preoperatively, all patients received oral celecoxib 200 mg twice a day. All patients received oral alprazolam 0.4 mg every night to aid in sleep and for antianxiety. During the operation, all patients received general anesthesia without the addition of any morphine or corticosteroids. The range of intraoperative blood pressures was controlled (approximately 90–100 mmHg). No tourniquet, urinary catheter, or drainage tube was used. Before placing the prosthesis, Group A (control group, 50 patients) received LIA with 200 mg ropivacaine with normal saline diluted to 80 ml (0.25% ropivacaine), and Group B received a cocktail LIA with 200 mg ropivacaine, 10 mg morphine, and 1 ml Diprospan (compound betamethasone injection containing 5 mg betamethasone dipropionate and 2 mg betamethasone sodium phosphate, calculated by betamethasone) that was combined with normal saline and diluted to 80 ml (0.25% ropivacaine, 0.125 mg/ml morphine, and 62.5 *μ*g/ml betamethasone). The LIA was injected into the following layers [6, 18], as shown in Figure 2: around the medial and lateral collateral ligament; the medial, lateral, and posterior capsules; the vastus medialis obliquus muscle and quadriceps tendon; and the prepatellar tissues and subcutaneous tissues. These site injections caused the following nerve endings that innervate the knee to be blocked, as shown in Figure 2: nerve to vastus intermedius (NVI), nerve to vastus lateralis (NVL), nerve to vastus medialis (NVM), lateral retinacular nerve from sciatic nerve (LRN), infrapatellar branch of saphenous nerve (IPN), and recurrent peroneal nerve from common peroneal nerve (RPN). Single-brand, posterior-stabilized, fixed-bearing, and multiradius prostheses (PFC, DePuy, Warsaw, IN, USA) were used. The prostheses were fixed with cement, and no patellar resurfacing was observed. All patients were given cephalosporin (1500 mg every 8 hours) for 24 hours to prevent infection. Low-molecular-weight heparin (0.4 ml) was administered for deep vein thrombosis. Oxycodone 10 mg or morphine 5 mg was used when the patients reported pain greater than 4 on a 0–10 visual analog scale (VAS). Continuous movement exercises were encouraged to help the patients recover postoperatively.

Preoperatively, demographic and baseline characteristics were collected, as listed in Table 1. The primary outcomes included pain, inflammatory markers, function, and sleeping quality. The pain evaluations were calculated by the resting or active pain VAS (postoperative (PO) 6 hours, 12 h, 24 h, 48 h, 72 h, 3 months, 6 m, 1 year, and 2 y) and opioid consumption (PO 1 d and 2 d). Opioid consumption was calculated by converting opioids consumed to morphine equivalents (MEs). The inflammatory markers included blood C-reactive protein (CRP) and interleukin-6 (IL-6, postoperative 1 day, 2 days, and 2 weeks) [19, 20]. The functional outcomes were knee range of motion (ROM, PO 1 d, 2 d, 3 d, 3 m, 6 m, 1 y, and 2 y), leg raising, and getting out of bed test (PO 1 d, 2 d, and 3 d). Sleep quality was measured by the Epworth Sleepiness Score [21] (ESS, PO 1 d and 2 d). The secondary outcomes included adverse events, satisfaction rate, hospital stay, knee HSS score (PO 6 m, 1 y, and 2 y), and SF-12 score (PO 6 m, 1 y, and 2 y) [22]. A four-point Likert scale (very satisfied, satisfied, normal, or dissatisfied) was utilized to record the satisfaction rate.

The required sample size was calculated based on the VAS score. A 1-point difference was defined as the minimum clinically important difference (MCID) based on a previous study where the average VAS difference from the clinical value was approximately 1 to 2 points [23]. A sample size of at least 23 in each group was required to reliably (with probability greater than 0.9, power = 0.9) detect an MCID ≥1, assuming a two-sided criterion for detection that allows for a maximum Type I error rate of *α* = 0.05. Thus, 50 patients in each group were required after considering dropout and withdrawal rates. Continuous variables such as CRP level, IL-6 level, pain VAS score, and ROM are presented as mean ± standard deviation, and independent-sample Student's *t* tests were used to calculate differences. Discontinuous variables such as the straight leg raise test and PONV rate are presented as frequencies (percentages), and Pearson *χ*^2^ tests or Fisher exact tests were used to calculate differences. The Kruskal‒Wallis H test was used to analyze ranked data such as satisfaction rate. All raw significance levels were set at *α* = 0.05, and *P* < 0.05 indicated a significant difference. All data were collected using Excel 2019 (Microsoft software), and statistical analyses were programmed and calculated using Jamovi 2.2 (retrieved from https://www.jamovi.org). The charts were drawn by GraphPad Prism 9.0 (GraphPad Software).

## 3. Results

A CONSORT flowchart of the procedure and participants is shown in Figure 1. A total of 142 patients were assessed for eligibility. Forty-two of those patients were excluded; 26 patients did not meet the inclusion criteria, and 16 patients refused to participate, as shown in Figure 1. There were no significant differences in terms of the characteristics between the two groups (*P* > 0.05). No patients in any of the groups were excluded from the analysis, as shown in Table 1.

### 3.1. Primary Outcomes

**Figure 3:**
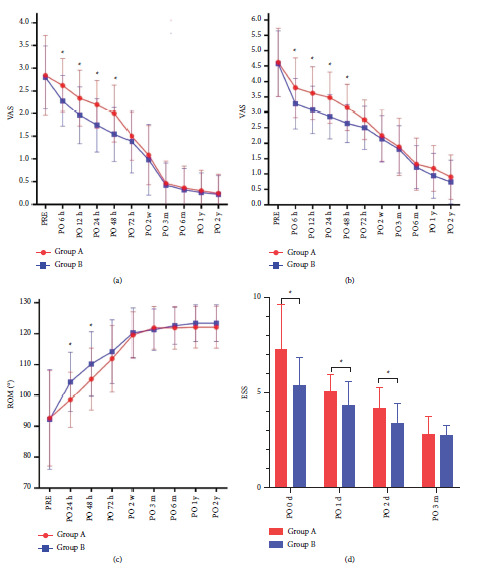
Primary outcomes: (a) VAS rest score; (b) VAS active score; (c) range of motion; (d) Epworth Sleepiness Score. ^*∗*^*P* < 0.05 indicates significant difference.

The primary outcomes are listed in Table 2 and Figure 3. For pain evaluation, Group A had significantly greater resting and active VAS pain scores than Group B within 48 h (*P* < 0.05). However, none of the between-group differences exceeded the MCID. After PO 48 h, no significant differences were observed through PO 2 y. Group A had more opioid consumption than Group B at PO 48 h (*P* < 0.001). For inflammatory markers, Group A had significantly lower levels of CRP and IL-6 than Group B at PO 48 h (*P* < 0.05). Regarding functional recovery, Group A had less ROM than Group B at PO 48 h, while there was no significant difference between the two groups after 48 h to 2 y. Nearly all patients could complete the straight leg raise and get out of bed tests at PO 48 h, although up to PO 24 h, Group B had a better degree of completion in the straight leg raise test. Group B had significantly lower ESS scores than Group A (*P* < 0.05), which indicated better sleep quality.

### 3.2. Secondary Outcomes

No significant differences were found in postoperative hospital stay. Group A had a significantly lower satisfaction rate for pain control than Group B, while the satisfaction rate for functional recovery was not significantly different. Regarding adverse events, Group B had slightly more patients with uroschesis than Group A. However, the two groups had no significant differences in PONV, uroschesis, or pruritus (*P* > 0.05). The patients with uroschesis were treated with urinary catheters. All adverse events resolved before discharge. No other adverse events, such as dizziness, hypotension, or wound infection, were observed. The HSS and SF-12 scores showed no significant differences at the 2-year follow-up. The secondary outcomes are listed in Table 3.

## 4. Discussion

The most important findings supported our hypotheses: adding morphine and betamethasone to LIA provided a longer-lasting analgesic effect. Although the pain differences did not exceed the MCID, the LIA cocktail reduced CRP and IL-6 levels, reduced opioid consumption up to PO 48 h, enhanced early recovery in terms of functional measures such as ROM, and improved sleeping quality, all without increasing the incidence of adverse events.

Pain is defined as an unpleasant feeling and emotional experience, accompanied by substantial or potential tissue damage or a description of these injuries [5]. Postoperative TKA pain leads to anxiety and insomnia and stimulates neuroendocrine responses, which have adverse effects on the development of chronic pain [8]. Chronic persistent sympathetic pain may result in joint fibrosis, long-term disability, impaired rehabilitation, and persistent dissatisfaction. Therefore, reasonable evaluation and treatment of pain can improve patient satisfaction and prevent pain from developing into chronic pain, which is also an important part of the ERAS management model of TKA [8]. Multimodal analgesia is an effective combination of different types of analgesic drugs and analgesic methods to relieve postoperative pain and reduce systemic adverse events. LIA is a method of injecting mixed analgesics into various layers of articular tissue during TKA, also known as cocktail analgesia. It is a novel method of intraoperative local analgesia that can effectively relieve postoperative pain and reduce adverse events. It has the advantages of high targeting, simplicity, and few systemic side effects, and its analgesic effect is remarkable [7]. However, at present, there is no unified standard for the formulation of local drugs. Moreover, the half-life of most anesthetics is less than 4 hours, and their postoperative analgesic effects are limited. Therefore, a combination of drugs, including opioids, long-acting local anesthetics, and epinephrine, is often used to increase the analgesic effect and prolong the analgesic duration.

LIA morphine can directly affect peripheral nerves that are injured or stimulated by traction, weaken the stimulation and conduction of pain, and achieve analgesia. Compared with oral or intravenous administration of opioids, LIA significantly reduces the use of parenteral anesthetics. A related study has shown that LIA morphine provides superior analgesic effects within the first 24 h compared to intrathecal morphine following total joint arthroplasty, and the risk of nausea [24], vomiting, and itching was also reduced. However, the action time of morphine alone is very short. Most studies show that the effect is significant 24 hours after the operation, while knee pain is present for a long time after TKA. Thus, postoperative pain can easily worsen, which can increase the total use of opioids after TKA. Moreover, the most common adverse reactions to morphine are mainly related to the digestive tract and central nervous system and include nausea, vomiting, constipation, lethargy, excessive sedation, and respiratory depression, which also affect the safety of morphine use.

Glucocorticoids inhibit the gene transcription of inflammatory cytokines, reduce the content of cyclooxygenase-2 (COX-2) and prostaglandin, inhibit the exudation of macrophages and other inflammatory cells, reduce the inflammatory reaction caused by macrophage activation, antagonize the 5-hydroxytryptamine (5-HT) receptor, inhibit the 5-HT-stimulated vomiting center, and alleviate nausea and vomiting caused by anesthesia and central drug stimulation [14, 16]. Betamethasone is a long-acting glucocorticoid that has a potency of approximately 20 times that of hydrocortisone and 5 times that of methylprednisolone without significant water and sodium retention. Diprospan, which contains betamethasone sodium phosphate and betamethasone dipropionate, was used in our study. Soluble betamethasone sodium phosphate is absorbed and takes effect quickly after injection. Betamethasone dipropionate is slightly soluble and acts as a reservoir for slow absorption, which slows the absorption of betamethasone and can play a lasting role, thus relieving symptoms for a long time. Glucocorticoids can theoretically reduce the central adverse effects of morphine, such as nausea and vomiting [25, 26]. In our study, the inflammatory markers CRP and IL-6 at 48 h after TKA were significantly lower in Group B than in Group A, and the pain score and morphine consumption within 48 h were lower in Group B than in Group A. Additionally, there was no difference in nausea, vomiting, or urine retention between the two groups. Moreover, no long-term complications were found during the 2-year follow-up. These results showed that betamethasone inhibits the inflammation caused by TKA and prolongs the duration of analgesia, which was beneficial to patient recovery after TKA.

Rebound pain is a temporary acute postoperative pain that occurs after the disappearance of regional anesthetic sensory blockade [27]. Rebound pain, as acute postoperative pain, can cause adverse effects; it often occurs at night, interfering with the patient's quality of sleep and seriously compromising recovery. Local infiltration injections of periarticular medications are also a type of regional block that may lead to postoperative transient rebound pain. The mechanism of rebound pain is unknown, and it may be relatively sudden nociceptive pain due to inadequate analgesia or hyperalgesia caused by regional blockade [28, 29]. Other factors may include neurotoxicity of the local anesthetic, withdrawal reactions, potential pain facilitation, and personal or surgical factors. Strategies to alleviate rebound pain include a multimodal analgesic regimen [30], patient education on appropriate expectations for postoperative pain, and timely use of analgesic medication. Prolonging the duration of action of regional anesthesia using local anesthetic adjuvants may also help to reduce rebound pain. It has been reported that the addition of adjuvants such as glucocorticoids [31] and acetaminophen [32] to local anesthesia can reduce the incidence of eruptive rebound pain and improve patient satisfaction. In our study, no rebound pain was found in patients after surgery. The addition of Diprospan to the LIA in this study allows for a sustained slow release of the drug, which can reduce local inflammation and maintain a good anti-inflammatory and analgesic effect after 48 hours postoperatively. Therefore, the formulation of ropivacaine, morphine, and Diprospan in our study is a good alternative to prolong analgesic effects after TKA.

Although betamethasone suppresses inflammation, relieves pain, and contributes to healing and recovery, the local use of steroids may delay wound healing and lead to adverse events such as wound infection [33, 34]. In our study, there were no wound complications in Group B. According to previous studies, there was no evidence of a significant increase in serious adverse events, such as skin necrosis and prolonged wound healing, after the use of glucocorticoids in TKA. Li et al.'s meta-analysis showed that the use of glucocorticoids in TKA did not increase the risk of infection or surgical healing complications [35]. However, the use of glucocorticoids in patients with high-risk complications for analgesia and anti-inflammation associated with TKA should be carefully considered.

There are also some limitations in our study. First, the betamethasone results do not apply to other types of glucocorticoids. Different glucocorticoids have different half-lives and anti-inflammatory effects. These results do not imply that other kinds of glucocorticoids have similar analgesic effects. Second, the resting and active pain differences between the two groups were below the MCID. This may be due to the application of multimodal analgesia since the postoperative patients showed mild symptoms. This may also be due to an insufficient sample size. Therefore, it is necessary to carry out a multicenter study with a larger sample size to confirm the effect more accurately.

## 5. Conclusions

A cocktail of ropivacaine, morphine, and Diprospan prolonged the analgesic effect to 48 h postoperatively. Although the small statistical benefit may not result in an MCID, the cocktail of ropivacaine, morphine, and Diprospan still reduces opioid consumption, provides better sleeping quality and faster rehabilitation, and does not increase the number of adverse events. Therefore, cocktails of ropivacaine, morphine, and Diprospan have good application value for pain control in TKA.

## Figures and Tables

**Table 1:** Baseline demographics and characteristics.

	Group A (*n* = 50)	Group B (*n* = 50)	*t*/*χ*^2^	*P*
Age	64.54 ± 4.55 (63.27 to 65.80)	64.44 ± 4.63 (63.16 to 65.72)	0.109	0.9135^a^
Gender (male/female)	13/37	12/38	0.053	0.817^b^
BMI (kg/m^2^)	24.17 ± 2.32 (23.52 to 24.80)	24.53 ± 1.15 (24.21 to 24.84)	0.983	0.328^a^
Comorbidities				
Hypertension	16 (32%)	14 (28%)	0.190	0.663^b^
Diabetes	10 (20%)	8 (16%)	0.271	0.603^b^
COPD	4 (8%)	5 (10%)	—	1.000^c^
Hypothyroidism	0 (0%)	1 (2%)	—	1.000^c^
Preoperative VAS				
Rest	2.84 ± 0.88 (2.59 to 3.08)	2.80 ± 0.69 (2.60 to 2.99)	0.253	0.801^a^
Activity	4.62 ± 1.11 (4.31 to 4.92)	4.58 ± 1.07 (4.28 to 4.87)	0.184	0.855^a^
Preoperative ROM	92.60 ± 15.47 (88.31 to 96.88)	92.20 ± 16.16 (87.72 to 96.67)	0.126	0.899^a^
Preoperative HSS	43.90 ± 8.63 (41.50 to 46.29)	45.50 ± 8.07 (43.26 to 47.73)	0.957	0.341^a^
ASA (I/II/III)	0/45/5	0/44/6	—	1.000^c^

BMI: body mass index; COPD: chronic obstructive pulmonary disease; ASA: American Society of Anesthesiologists. ^a^The *P* value represented the result of Student's *t* test for continuous variables between 2 groups. ^b^The *P* value represented the result of Pearson's *χ*^2^ test for discontinuous variables between 2 groups. ^c^The *P* value represented the result of Fisher's exact test for discontinuous variables between 2 groups. *P* < 0.05 indicated significant differences.

**Table 2:** Primary outcomes.

	Group A (*n* = 50)	Group B (*n* = 50)	*t*/*χ*^2^	*P*
Inflammatory indicator				
CRP				
Pre	4.25 ± 0.89 (4.00 to 4.49)	4.35 ± 0.98 (4.07 to 4.62)	0.534	0.595
PO 24 h	54.60 ± 12.76 (51.06 to 58.13)	44.22 ± 12.34 (40.79 to 47.64)	4.135	**<0.001** ^*∗*^^**a**^
PO 48 h	65.95 ± 14.41 (61.95 to 69.94)	59.49 ± 13.01 (55.88 to 63.09)	2.320	**0.022** ^*∗*^^**a**^
PO 14 d	4.28 ± 0.84 (4.04 to 4.51)	4.26 ± 0.86 (4.02 to 4.49)	0.117	0.906
IL-6				
Pre	8.01 ± 4.11 (6.87 to 9.14)	7.96 ± 3.84 (6.57 to 8.72)	0.063	0.063
PO 24 h	84.19 ± 17.01 (79.47 to 88.90)	57.42 ± 12.46 (53.96 to 60.87)	8.977	**<0.001** ^*∗*^^**a**^
PO 48 h	60.72 ± 15.49 (56.42 to 65.01)	44.11 ± 13.67 (40.32 to 47.89)	5.685	**<0.001** ^*∗*^^**a**^
PO 14 d	7.89 ± 4.17 (6.73 to 9.04)	7.65 ± 3.89 (6.57 to 8.72)	0.297	0.766
Pain				
VAS rest				
PO 6 h	2.62 ± 0.59 (2.45 to 2.78)	2.28 ± 0.56 (2.12 to 2.43)	2.955	**0.004** ^*∗*^^**a**^
PO 12 h	2.34 ± 0.62 (2.16 to 2.51)	1.96 ± 0.63 (1.78 to 2.13)	3.119	**0.002** ^*∗*^^**a**^
PO 24 h	2.20 ± 0.53 (1.82 to 2.17)	1.74 ± 0.59 (1.57 to 1.90)	4.101	**<0.001** ^*∗*^^**a**^
PO 48 h	2.00 ± 0.63 (2.05 to 2.34)	1.54 ± 0.60 (1.37 to 1.70)	3.738	**<0.001** ^*∗*^^**a**^
PO 72 h	1.50 ± 0.53 (1.35 to 1.64)	1.38 ± 0.69 (1.18 to 1.57)	0.975	0.332^a^
PO 2 w	1.08 ± 0.65 (0.89 to 1.26)	0.98 ± 0.78 (0.76 to 1.19)	0.905	0.367^a^
PO 3 m	0.46 ± 0.49 (0.32 to 0.59)	0.42 ± 0.49 (0.28 to 0.55)	0.408	0.684^a^
PO 6 m	0.36 ± 0.48 (0.22 to 0.49)	0.32 ± 0.47 (0.18 to 0.45)	0.421	0.675^a^
PO 1 y	0.30 ± 0.45 (0.17 to 0.42)	0.26 ± 0.43 (0.14 to 0.37)	0.454	0.651^a^
PO 2 y	0.24 ± 0.42 (0.12 to 0.35)	0.22 ± 0.41 (0.10 to 0.33)	0.241	0.810^a^
VAS activity				
PO 6 h	3.80 ± 0.97 (3.53 to 4.06)	3.28 ± 0.82 (3.05 to 3.50)	2.895	**0.004** ^*∗*^^**a**^
PO 12 h	3.62 ± 0.86 (3.38 to 3.85)	3.08 ± 0.77 (2.86 to 3.29)	3.307	**0.001** ^*∗*^^**a**^
PO 24 h	3.48 ± 0.83 (3.24 to 3.71)	2.86 ± 0.72 (2.66 to 3.05)	3.989	**<0.001** ^*∗*^^**a**^
PO 48 h	3.16 ± 0.75 (2.95 to 3.36)	2.64 ± 0.62 (2.46 to 2.81)	3.778	**<0.001** ^*∗*^^**a**^
PO 72 h	2.76 ± 0.64 (2.58 to 2.93)	2.50 ± 0.70 (2.30 to 2.69)	1.938	0.055^a^
PO 2 w	2.24 ± 0.83 (2.00 to 2.47)	2.14 ± 0.75 (1.93 to 2.34)	0.600	0.533^a^
PO 3 m	1.88 ± 0.93 (1.62 to 2.13)	1.80 ± 0.77 (1.58 to 2.01)	0.468	0.640^a^
PO 6 m	1.32 ± 0.85 (1.08 to 1.55)	1.22 ± 0.70 (1.02 to 1.41)	0.412	0.522^a^
PO 1 y	1.18 ± 0.74 (0.97 to 1.38)	0.94 ± 0.73 (0.73 to 1.14)	1.632	0.106^a^
PO 2 y	0.90 ± 0.72 (0.70 to 1.09)	0.74 ± 0.71 (0.54 to 0.93)	1.118	0.265^a^
Opioid consumption				
PO 24 h (mg)	6.75 ± 7.23 (4.74 to 8.75)	3.70 ± 5.41 (2.20 to 5.19)	2.388	**0.018** ^*∗*^^**a**^
PO 72 h (mg)	13.80 ± 14.68 (9.73 to 17.86)	7.60 ± 11.10 (4.52 to 10.67)	2.382	**0.019** ^*∗*^^**a**^
Function				
ROM				
PO 24 h	98.60 ± 8.94 (96.12 to 101.08)	104.40 ± 9.62 (101.73 to 107.06)	3.122	**0.002** ^*∗*^^**a**^
PO 48 h	105.30 ± 10.02 (102.52 to 108.07)	110.20 ± 10.46 (107.30 to 113.09)	2.392	**0.018** ^*∗*^^**a**^
PO 72 h	111.90 ± 10.72 (108.92 to 114.87)	114.20 ± 10.31 (111.34 to 117.05)	1.093	0.277^a^
PO 2 w	119.60 ± 7.53 (117.51 to 121.68)	120.30 ± 8.08 (118.06 to 122.53)	0.491	0.624^a^
PO 3 m	121.90 ± 6.92 (119.98 to 123.81)	121.30 ± 6.69 (119.44 to 123.15)	0.441	0.660^a^
PO 6 m	121.90 ± 6.92 (119.98 to 123.81)	122.60 ± 6.01 (120.93 to 124.26)	0.540	0.590^a^
PO 1 y	122.10 ± 6.78 (119.22 to 122.97)	123.40 ± 5.95 (121.75 to 125.04)	1.019	0.311^a^
PO 2 y	122.20 ± 6.79 (120.31 to 124.08)	123.40 ± 5.95 (121.75 to 125.04)	0.939	0.349^a^
Straight leg raise test				
PO 24 h	32 (64%)	46 (92%)	11.4	**<0.001** ^b^
PO 48 h	48 (96%)	49 (98%)	—	1.000^c^
Discharge	50 (100%)	50 (100%)	—	1.000^c^
Get out of bed and walk test				
PO 24 h	45 (90%)	46 (92%)	0.122	0.727^b^
PO 48 h	48 (96%)	49 (98%)	—	1.000^c^
Discharge	50 (100%)	50 (100%)	—	1.000^c^
Epworth Sleepiness Score				
PO 0 d	7.26 ± 2.39 (6.59 to 7.92)	5.38 ± 1.44 (4.98 to 5.77)	4.764	**<0.001** ^*∗*^^**a**^
PO 1 d	5.10 ± 0.83 (4.86 to 5.33)	4.36 ± 1.26 (4.01 to 4.70)	3.468	**<0.001** ^*∗*^^**a**^
PO 2 d	4.20 ± 1.06 (3.90 to 4.49)	3.40 ± 1.03 (3.11 to 3.68)	3.827	**<0.001** ^*∗*^^**a**^
PO 3 m	2.82 ± 0.91 (2.56 to 3.07)	2.76 ± 0.55 (2.60 to 2.91)	0.398	0.691^a^

VAS: visual analog scale; ROM: range of motion; PO: postoperative; h: hour; d: day; w: week; m: month; y: year. ^a^The *P* value represented the result of Student's *t* test for continuous variables between 2 groups. ^b^The *P* value represented the result of Pearson's *χ*^2^ test for discontinuous variables between 2 groups. ^c^The *P* value represented the result of Fisher's exact test for discontinuous variables between 2 groups. ^∗^ and bold values mean that *P* < 0.05 indicates significant differences.

**Table 3:** Secondary outcomes.

	Group A (*n* = 50)	Group B (*n* = 50)	*t*/*H*	*P*
Pain control			5.410	**0.020** ^**a**^
Very satisfied	25 (50%)	36 (72%)		
Satisfied	15(30%)	10 (20%)		
Normal	10 (20%)	4 (8%)		
Dissatisfied	0 (0%)	0 (0%)		
Function recovery			0.172	0.678^a^
Very satisfied	35 (70%)	36 (72%)		
Satisfied	14 (28%)	14 (28%)		
Normal	1 (2%)	0 (0%)		
Dissatisfied	0 (0%)	0 (0%)		
Hospital stays (d)	2.96 ± 0.56	2.74 ± 0.55	1.981	0.050^b^
HSS				
PO 6 m	87.68 ± 6.22	88.30 ± 4.92	0.553	0.582^b^
PO 1 y	89.60 ± 5.28	90.30 ± 4.92	0.685	0.494^b^
PO 2 y	90.88 ± 4.23	91.20 ± 3.96	0.391	0.697^b^
SF-12 (PCS)				
PO 6 m	21.66 ± 3.17	21.74 ± 3.41	0.122	0.904^b^
PO 1 y	22.54 ± 3.18	22.86 ± 3.32	0.492	0.624^b^
PO 2 y	23.50 ± 3.22	23.36 ± 3.34	0.213	0.831^b^
SF-12 (MCS)				
PO 6 m	24.00 ± 3.04	24.20 ± 3.20	0.320	0.749^b^
PO 1 y	25.84 ± 2.85	25.82 ± 3.30	0.033	0.974^b^
PO 2 y	26.42 ± 2.09	26.32 ± 2.94	0.196	0.845^b^
Adverse events				
Wound infections	0	0	—	—
Skin itch	0	0	—	—
PONV	3 (6%)	3 (6%)	—	1.000^c^
Urinary retention	2 (4%)	3 (6%)	—	1.000^c^
Acute myocardial infarction	0	0	—	—
Stroke	0	0	—	—
Acute renal failure	0	0	—	—
Acute liver failure	0	0	—	—
Deep venous thrombosis	0	0	—	—
Pulmonary embolism	0	0	—	—

HSS: hospital for special surgery score; SF-12: 12 short form scale; PCS: Physical Component Summary; MCS: Mental Component Summary; PO: postoperative; m: month; y: year. ^a^The *P* value represented the result of the Kruskal–Wallis *H* test for ranked data between 2 groups. ^b^The *P* value represented the result of Student's *t* test for continuous variables between 2 groups. ^c^The *P* value represented the result of Fisher's exact test for discontinuous variables between 2 groups. The bold value means that *P* < 0.05 indicates significant differences.

## Data Availability

All detailed data have been listed in tables and supplementary files uploaded.

## Supplementary Materials

Supplementary Materials File 1: collecting raw data of Group A (control group). File 2: collecting raw data of Group B (LIA group). File 3: ICMJE Form for Disclosure of Potential Conflicts of Interest.
