# Supplementary material for: Cocktail of Ropivacaine, Morphine, and Diprospan Reduces Pain and Prolongs Analgesic Effects after Total Knee Arthroplasty: A Prospective Randomized Controlled Trial
Source: Int J Clin Pract. 2024 Feb 28;2024:3697846. doi: 10.1155/2024/3697846 (PMC10917473; doi:10.1155/2024/3697846)
Supplement: Supplementary Materials — File 1: collecting raw data of Group A (control group). File 2: collecting raw data of Group B (LIA group). File 3: ICMJE Form for Disclosure of Potential Conflicts of Interest. [file 3697846.f1.zip › File 1_ Collecting Raw data-Group A (Control).pdf]

Patients number	Randomized number	Age	Gender	Height （cm）	Weight （Kg）	BMI (kg/m2)	Patients			IL-6		
							PRE	POD 1 D	POD 2 D	PRE	POD 1 D	POD 2 D
1	35	64	Male	166	65	23.5883292	5.36	48.28	60.77	2.08	94.94	65.78
2	27	61	Female	157	60	24.3417583	3.66	42.22	51.76	2.85	92.58	64.36
3	39	64	Female	157	55	22.3132784	5.06	39.15	47.61	7.77	99.92	74.52
4	43	58	Male	172	72	24.3374797	6.76	63.03	77.62	7.43	97.9	69.32
5	47	63	Male	172	71	23.9994592	3.09	72.39	79.18	4.13	98.62	73.12
6	9	64	Female	159	61	24.1287924	3.8	59.23	74.8	8.11	54.56	35.62
7	21	65	Female	163	58	21.8299522	3.54	60.94	74.98	17.47	70.3	51.37
8	51	67	Female	150	75	33.3333333	3.59	62.82	75.73	7.56	98.26	71.36
9	39	62	Female	160	65	25.390625	3.25	57.51	73.69	11.81	94.39	64.57
10	45	63	Female	160	55	21.484375	4.15	46.85	60.39	8.03	102.21	84.99
11	7	67	Female	165	75	27.5482094	3.52	74.68	86.6	4.24	59.78	35.58
12	9	64	Male	175	74	24.1632653	4.85	46.62	60.16	1.5	63.84	42.16
13	43	69	Male	170	70	24.2214533	4.78	54.31	70.39	9.21	71.65	53.18
14	15	59	Female	165	68	24.9770432	4.11	54.15	67.59	3.29	126.57	93.79
15	41	64	Female	163	66	24.8409801	4.86	62.47	75.56	4.47	83.03	58.78
16	41	70	Female	160	62	24.21875	5.64	33.73	34.61	1.5	91.98	63.74
17	67	70	Female	154	53	22.3477821	4.22	68.89	78.54	5.31	55.04	31.19
18	45	64	Male	178	76	23.9868703	4.32	53.15	66.86	7.14	105.28	86.08
19	33	66	Female	160	65	25.390625	3.64	57.99	74.49	14.77	73.26	54.59
20	65	69	Female	155	56	23.3090531	4.48	30.6	31.4	15.43	75.36	56.13
21	59	66	Female	162	58	22.1002896	4.79	74.18	82.57	3.38	106.52	87.47
22	55	63	Female	164	67	24.9107674	3.94	45.83	59.01	9.01	69.49	50.17
23	67	62	Female	151	65	28.5075216	4.91	57.66	73.74	8.81	67.03	44.96
24	55	61	Male	174	72	23.7812128	4.02	86.23	99.81	1.5	84.29	59.84
25	49	78	Female	156	55	22.600263	3.61	74.06	79.77	11.79	106.96	89.03
26	37	66	Female	150	52	23.1111111	4.36	41.61	50.47	8.97	122.66	91.63
27	47	71	Female	163	66	24.8409801	3.98	52.94	66.25	2.89	88.95	62.03
28	41	63	Female	157	59	23.9360623	4.6	45.04	55.48	4.54	76.09	57.5
29	5	67	Female	155	56	23.3090531	6.57	54.41	70.48	15.93	63.56	41.5
30	33	53	Female	152	52	22.5069252	3.35	53.53	67.37	12.73	87.25	61.17
31	23	71	Female	163	65	24.4646016	4.78	53.68	67.52	9.74	71.55	51.55
32	23	61	Male	182	75	22.642193	4.45	34.11	34.66	9.63	74.77	56.12
33	59	69	Female	158	60	24.0346098	4.24	76.9	87.69	6.57	82.41	57.67
34	53	63	Male	175	73	23.8367347	2.86	49.35	62.81	3.32	76.66	57.53
35	41	72	Female	165	67	24.6097337	3.9	54.16	67.97	7.89	101.64	75.47
36	47	57	Female	150	52	23.1111111	3.51	44.81	54.96	13.06	91.54	63.18
37	59	62	Female	155	55	22.89282	5.14	46.32	59.58	13.65	98.33	71.46
38	55	63	Female	156	58	23.8330046	3.45	51.28	63.28	7.64	87.54	61.66
39	15	65	Female	145	45	21.4030916	4.15	45.82	56.02	6.39	62.31	37.31
40	13	58	Male	176	75	24.2122934	5.15	48.69	61.57	8.06	100.88	74.97
41	45	71	Female	160	61	23.828125	5.07	81.39	89.76	5.01	58.18	32.52
42	83	60	Male	175	80	26.122449	5.16	40.19	47.94	15.17	74.56	55.15
43	63	65	Female	166	60	21.7738424	5.53	40.79	49.6	15.56	72.74	53.54
44	61	70	Female	160	61	23.828125	2.62	62.97	77.57	6.39	65	43.82
45	67	61	Female	155	55	22.89282	2.79	57.3	72.97	6.8	67.86	47.29
46	7	64	Female	155	54	22.4765869	3.96	38.4	47.41	6.91	88.44	61.82
47	21	68	Female	160	55	21.484375	4.6	67.36	77.99	9.67	69.61	51.23
48	41	65	Female	150	75	33.3333333	3.78	52.31	63.82	9.46	97.7	65.98
49	43	56	Male	176	75	24.2122934	4.26	69.15	78.96	7.88	101.76	83.11
50	59	63	Female	162	64	24.3865264	2.56	40.71	48.06	8.54	83.89	59.1
mean		64.54		161.78	63.38	24.1746854	4.2554	54.6038	65.9564	8.0198	84.1928	60.7202
std		4.55284526		8.51185056	8.23137899	2.32507435	0.89479877	12.7673429	14.407793	4.11540884	17.0105019	15.49328

Patients number	VAS Rest											
	PRE	POD 6 H	POD 12 H	POD 24 H	POD 48 H	POD 72 H	POD 2W	POD 3 M	POD 6 M	POD 1 Y	POD 2 Y	PRE	POD 6 H
1	2	4	3	3	3	2	2	1	1	1	1	4	6
2	3	3	3	3	3	2	2	1	1	1	1	5	5
3	4	2	2	2	2	1	1	0	0	0	0	6	3
4	3	2	2	2	2	1	1	0	0	0	0	4	3
5	4	3	3	3	3	2	2	1	1	1	1	4	5
6	2	3	3	3	3	2	2	1	1	1	1	4	5
7	1	3	3	3	3	2	2	1	1	1	1	4	5
8	4	2	2	2	2	1	1	0	0	0	0	6	3
9	4	2	2	2	2	1	1	0	0	0	0	6	3
10	3	3	3	3	3	2	2	1	1	1	1	4	5
11	3	4	4	3	3	2	2	1	1	1	1	3	6
12	3	3	3	3	3	2	2	1	1	1	1	6	5
13	3	3	3	3	3	2	2	1	1	1	1	2	5
14	3	2	2	2	2	1	1	0	0	0	0	3	3
15	3	3	3	3	2	2	2	1	1	1	1	5	5
16	2	2	2	2	2	1	1	0	0	0	0	5	3
17	4	2	2	2	2	1	1	0	0	0	0	6	3
18	1	2	2	2	2	1	1	0	0	0	0	4	3
19	3	3	3	3	2	2	2	1	1	1	1	4	5
20	3	3	3	3	2	2	2	1	1	1	0	3	5
21	3	3	3	2	2	2	1	1	1	1	0	5	5
22	4	4	4	3	3	3	2	1	1	1	1	6	5
23	2	2	2	2	2	1	1	0	0	0	0	6	3
24	2	3	3	2	2	2	1	1	1	1	0	6	4
25	3	3	3	2	2	2	1	1	1	0	0	4	4
26	1	2	2	2	2	1	1	0	0	0	0	3	3
27	4	3	3	2	2	2	1	1	1	0	0	4	4
28	4	3	2	2	2	2	1	1	1	0	0	4	4
29	2	2	2	2	2	1	1	0	0	0	0	5	3
30	1	2	2	2	2	1	1	0	0	0	0	6	3
31	3	3	2	2	2	2	1	1	0	0	0	4	4
32	3	3	2	2	2	2	1	1	0	0	0	7	4
33	2	2	2	2	1	1	1	0	0	0	0	6	3
34	3	3	2	2	2	2	1	1	0	0	0	5	4
35	3	2	2	2	1	1	0	0	0	0	0	4	3
36	3	3	2	2	2	2	1	1	0	0	0	4	4
37	4	3	2	2	2	2	1	1	0	0	0	5	4
38	2	3	2	2	2	2	1	0	0	0	0	6	4
39	1	2	2	2	1	1	0	0	0	0	0	4	3
40	3	2	2	2	1	1	0	0	0	0	0	4	3
41	3	2	2	2	1	1	0	0	0	0	0	4	3
42	3	2	2	2	1	1	0	0	0	0	0	6	3
43	4	3	2	2	2	1	1	0	0	0	0	5	4
44	3	2	2	2	1	1	0	0	0	0	0	5	3
45	3	3	2	2	2	1	1	0	0	0	0	4	4
46	3	3	2	2	2	1	1	0	0	0	0	5	3
47	3	2	2	1	1	1	0	0	0	0	0	4	3
48	2	2	1	1	1	1	0	0	0	0	0	6	2
49	3	2	1	1	1	1	0	0	0	0	0	3	2
50	4	3	2	2	2	1	1	0	0	0	0	3	3
mean	2.84	2.62	2.34	2.2	2	1.5	1.08	0.46	0.36	0.3	0.24	4.62	3.8
std	0.88	0.59632206	0.62	0.52915026	0.63245553	0.53851648	0.65848311	0.49839743	0.48	0.45825757	0.42708313	1.11157546	0.9797959

Patients number	VAS Active									Opioid consumption		
	POD 12 H	POD 24 H	POD 48 H	POD 72 H	POD 2W	POD 3 M	POD 6 M	POD 1 Y	POD 2 Y	POD 1 D	POD 3 D	PRE
1	6	5	5	4	4	3	3	2	2	20	40	120
2	5	5	5	4	3	3	3	2	2	15	30	90
3	3	3	3	3	2	2	1	1	1	0	0	90
4	3	3	3	3	2	2	1	1	1	0	0	80
5	5	5	5	4	3	3	3	2	2	20	40	100
6	5	5	4	4	3	3	2	2	2	20	40	80
7	5	5	4	3	3	3	2	2	2	20	40	110
8	3	3	3	3	2	2	1	1	1	0	0	80
9	3	3	3	3	2	1	1	1	1	0	0	120
10	5	4	4	3	3	3	2	2	2	10	20	110
11	5	5	5	4	4	3	3	2	2	20	40	120
12	5	4	4	3	3	3	2	2	2	20	40	90
13	4	4	3	3	3	3	2	2	2	15	30	90
14	3	3	3	2	2	1	1	1	1	0	0	100
15	4	4	3	3	3	3	2	2	2	10	20	90
16	3	3	3	2	2	1	1	1	1	0	0	90
17	3	3	3	2	2	1	1	1	0	0	0	70
18	3	3	3	2	2	1	1	1	0	0	0	80
19	4	4	3	3	3	3	2	2	1	15	30	90
20	4	4	3	3	3	3	2	2	1	12.5	30	120
21	4	4	3	3	3	3	2	2	1	15	30	90
22	5	5	5	4	4	3	3	2	2	10	20	100
23	3	3	3	2	2	1	1	1	0	0	0	80
24	4	4	3	3	3	3	2	2	1	5	10	80
25	4	4	3	3	3	3	2	2	1	7.5	20	80
26	3	3	3	2	2	1	1	1	0	0	0	120
27	4	4	3	3	3	2	2	2	1	12.5	30	100
28	4	4	3	3	3	2	2	2	1	10	20	110
29	3	3	3	2	2	1	1	1	0	0	0	80
30	3	3	3	2	2	1	1	1	0	0	0	80
31	4	4	3	3	3	2	2	2	1	10	20	80
32	4	4	3	3	2	2	1	1	1	10	20	80
33	3	3	3	2	1	1	1	0	0	0	0	100
34	4	4	3	3	2	2	1	1	1	10	20	90
35	3	3	3	2	1	1	1	0	0	0	0	80
36	4	3	3	3	2	2	1	1	1	10	20	80
37	4	3	3	3	2	2	1	1	1	10	20	80
38	3	3	3	3	2	2	1	1	1	10	20	90
39	3	3	3	2	1	1	0	0	0	0	0	80
40	3	3	3	2	1	1	0	0	0	0	0	120
41	3	3	2	2	1	1	0	0	0	0	0	70
42	3	3	2	2	1	1	0	0	0	0	0	120
43	3	3	3	3	2	2	1	1	1	10	20	120
44	3	2	2	2	1	1	0	0	0	0	0	80
45	3	3	3	3	2	2	1	1	1	10	20	110
46	3	3	3	3	2	2	1	1	1	0	0	90
47	3	2	2	2	1	0	0	0	0	0	0	80
48	2	2	2	2	1	0	0	0	0	0	0	70
49	2	2	2	2	1	0	0	0	0	0	0	80
50	3	3	3	3	2	2	1	1	1	0	0	90
mean	3.62	3.48	3.16	2.76	2.24	1.88	1.32	1.18	0.9	6.75	13.8	92.6
std	0.86925255	0.83042158	0.75789181	0.64992307	0.83809307	0.93037627	0.85883642	0.74	0.72801099	7.23273807	14.68196172	15.467385

Patients number	ROM								EES				Hospital stay
	**POD 1 D**	**POD 2 D**	**POD 3 D**	**POD 2W**	**POD 3 M**	**POD 6 M**	**POD 1 Y**	**POD 2 Y**	**POD 0 D**	**POD 1 D**	**POD 2 D**	**POD 3M**	（day）
1	120	120	130	130	130	130	130	130	11	6	6	4	4
2	100	100	110	120	120	120	125	125	7	5	4	2	3
3	100	110	120	120	125	125	125	125	8	5	4	2	4
4	90	100	100	110	120	120	120	120	5	5	4	3	3
5	100	110	120	125	125	125	125	125	9	5	5	2	3
6	90	100	100	110	120	120	120	120	4	4	3	4	3
7	100	120	120	130	130	130	130	130	10	6	5	3	3
8	90	100	100	110	110	110	115	115	4	4	3	2	3
9	120	125	130	130	130	130	130	130	11	7	6	3	3
10	100	110	120	125	130	130	130	130	10	6	5	4	4
11	120	120	130	130	130	130	130	130	11	7	6	2	3
12	100	100	110	120	120	120	120	120	7	5	4	3	3
13	100	100	110	120	120	120	120	120	7	5	4	3	2
14	100	110	120	125	125	125	125	125	9	5	5	4	3
15	100	110	110	120	125	125	125	125	8	5	4	1	3
16	100	100	110	120	120	120	120	125	7	5	4	3	3
17	80	90	100	110	110	110	110	110	4	4	2	3	3
18	100	100	110	120	120	120	120	120	7	5	4	2	4
19	100	110	120	120	125	125	125	125	9	5	5	4	3
20	110	120	130	130	130	130	130	130	11	6	6	3	2
21	100	100	110	120	120	120	120	120	7	5	4	3	2
22	100	110	120	125	130	130	130	130	9	6	5	5	4
23	100	100	100	120	120	120	120	120	6	5	4	2	3
24	90	100	100	110	120	120	120	120	5	4	3	2	3
25	100	100	100	120	120	120	120	120	6	5	4	2	3
26	110	120	130	130	130	130	130	130	11	6	6	3	4
27	100	110	120	125	130	130	130	130	9	6	5	3	3
28	100	120	125	130	130	130	130	130	10	6	5	4	2
29	90	100	100	110	120	120	120	120	5	5	4	4	3
30	90	100	100	110	120	120	120	120	5	5	4	4	2
31	100	100	110	120	120	120	120	120	7	5	4	2	3
32	100	100	110	120	120	120	120	120	7	5	4	3	3
33	100	110	120	125	130	130	130	130	9	6	5	4	2
34	100	100	110	120	120	120	120	120	7	5	4	3	3
35	90	100	100	110	110	110	110	110	4	4	3	1	3
36	90	90	100	110	110	110	110	110	4	4	3	2	2
37	90	90	100	110	110	110	110	110	4	4	3	2	3
38	100	110	120	120	125	125	125	125	8	5	5	2	3
39	100	100	100	110	120	120	120	120	6	5	4	4	3
40	110	120	125	130	130	130	130	130	10	6	6	3	3
41	80	90	100	110	110	110	110	110	4	4	3	3	3
42	110	120	125	130	130	130	130	130	10	6	6	2	3
43	110	120	125	130	130	130	130	130	10	6	6	3	3
44	90	100	100	110	120	120	120	120	5	5	4	3	3
45	100	120	125	130	130	130	130	130	10	6	5	3	3
46	100	100	110	120	120	120	120	120	7	5	4	2	3
47	90	90	100	110	110	110	110	110	4	4	3	2	2
48	80	90	100	110	110	110	110	110	3	3	2	4	4
49	90	90	100	110	110	110	110	110	4	4	3	3	2
50	100	110	110	120	125	125	125	125	8	5	4	1	3
mean	98.6	105.3	111.9		121.9	121.9	122.1	122.2	7.26	5.1	4.28	2.82	2.96
std	8.9465077	10.020479	10.7186753		6.92026011	6.92026011	6.78896163	6.7941151	2.39841614	0.83066239	1.05905618	0.90972523	0.56426944

Patients number		HSS			PCS			MCS		
	PRE	POD 6M	POD 1 Y	POD 2 Y	POD 6M	POD 1 Y	POD 2 Y	POD 6M	POD 1 Y	POD 2 Y
1	32	77	82	84	16	17	18	18	20	24
2	42	86	88	90	22	23	24	24	26	26
3	39	86	88	90	20	21	22	23	25	25
4	50	92	93	95	24	24	25	26	28	28
5	38	84	86	88	19	20	21	23	25	25
6	55	96	96	95	25	26	27	28	29	29
7	36	82	85	87	19	19	20	21	23	25
8	55	96	96	95	25	26	27	28	29	29
9	36	75	80	85	16	17	18	18	20	22
10	36	82	85	87	19	19	20	22	24	25
11	33	77	80	82	16	17	18	18	20	22
12	44	88	90	92	22	23	24	24	26	26
13	44	89	91	93	22	23	24	24	26	26
14	38	84	86	88	20	21	22	23	25	25
15	39	86	88	90	20	22	23	23	25	25
16	43	87	89	91	22	23	24	24	26	26
17	57	97	98	98	27	28	29	28	30	30
18	45	89	91	93	23	24	25	25	27	27
19	38	85	87	89	20	21	22	23	25	25
20	32	78	82	84	17	18	19	19	21	24
21	44	89	91	93	23	24	25	25	27	27
22	37	83	85	87	19	20	20	22	24	25
23	50	90	92	94	23	24	25	26	28	28
24	55	94	96	95	24	25	26	27	29	29
25	50	90	92	94	23	24	25	26	28	28
26	32	80	83	85	17	18	19	20	22	24
27	37	83	85	87	19	20	21	23	25	25
28	33	82	84	86	19	19	20	21	23	25
29	50	93	94	95	24	24	25	26	28	28
30	50	93	95	95	24	24	25	27	28	28
31	50	89	91	93	23	24	25	25	27	27
32	45	89	91	93	23	24	25	25	27	27
33	37	83	85	87	19	20	20	22	24	25
34	43	87	89	91	22	23	24	24	26	26
35	55	96	96	95	25	26	27	28	29	29
36	56	97	96	95	26	27	28	28	29	29
37	57	97	98	95	27	28	29	28	30	30
38	38	85	87	89	20	21	22	23	25	25
39	50	90	92	94	24	24	25	26	28	28
40	32	80	83	85	18	19	20	20	22	24
41	57	97	98	98	27	28	29	28	30	30
42	32	81	83	85	18	19	20	20	22	24
43	32	81	84	86	18	19	20	20	22	24
44	53	93	95	95	24	25	26	27	28	28
45	33	81	84	86	19	19	20	20	22	25
46	44	88	90	92	22	23	24	24	26	26
47	57	97	98	95	26	27	28	28	29	29
48	57	98	98	98	27	28	29	28	30	30
49	56	96	96	95	26	27	28	28	29	29
50	41	86	88	90	20	22	23	23	25	25
mean	43.9	87.68	89.6	90.88	21.66	22.54	23.5	24	25.84	26.42
std	8.63307593	6.22395373	5.28393793	4.23150092	3.16613329	3.18251473	3.22024844	3.03973683	2.85909076	2.09847564
