# Supplementary material for: Cocktail of Ropivacaine, Morphine, and Diprospan Reduces Pain and Prolongs Analgesic Effects after Total Knee Arthroplasty: A Prospective Randomized Controlled Trial
Source: Int J Clin Pract. 2024 Feb 28;2024:3697846. doi: 10.1155/2024/3697846 (PMC10917473; doi:10.1155/2024/3697846)
Supplement: Supplementary Materials — File 1: collecting raw data of Group A (control group). File 2: collecting raw data of Group B (LIA group). File 3: ICMJE Form for Disclosure of Potential Conflicts of Interest. [file 3697846.f1.zip › File 2_Collecting Raw data-Group B (LIA M+B).pdf]

Patients number	Randomized number	Age	Gender	（cm）	（Kg）	BMI (kg/m2)	CRP			IL-6			
							PRE	POD 1 D	POD 2 D	PRE	POD 1 D	POD 2 D	PRE
1	36	73	Female	160	65	25.390625	5.35	52.89	70.35	5.13	53.87	40.41	2
2	42	68	Female	158	60	24.0346098	5.29	59.43	75.78	10.32	55.64	42.77	3
3	42	64	Female	150	55	24.4444444	3.81	66.3	86.04	1.5	60.03	46.1	3
4	62	67	Female	160	64	25	2.98	63.8	79.64	1.5	48.58	35.53	3
5	12	73	Female	155	59	24.5577523	3.33	54.9	70.5	12.62	47.3	33.97	2
6	52	64	Female	152	57	24.6710526	4.84	40.79	58.34	6.25	44.63	30.39	4
7	64	68	Male	166	69	25.0399187	3.44	31.37	47.57	9.59	48.75	35.58	3
8	80	62	Female	162	67	25.5296449	5.96	54.93	62.48	8.01	57.29	44.24	3
9	58	64	Female	162	66	25.1486054	3.57	59.35	75.15	15.51	63.95	50.17	4
10	16	69	Female	165	68	24.9770432	4.54	51.38	70.09	8.49	77.56	63.99	2
11	36	63	Female	151	56	24.5603263	4.12	27.39	40.29	5.92	63.31	49.25	4
12	62	66	Female	150	54	24	3.68	67.15	90.15	6.09	84.5	77.39	3
13	40	67	Male	170	72	24.9134948	4.27	26.15	35.94	6.57	79.31	66.57	2
14	52	62	Female	160	64	25	2.87	27.42	41.03	10.73	46.32	32.32	3
15	16	63	Female	149	52	23.4223684	3.7	33.43	54.8	6.31	68.69	57.37	4
16	44	57	Male	167	69	24.7409373	5.98	32.44	48.42	1.5	64.01	50.56	3
17	30	63	Female	147	65	30.0800592	3.99	61.4	76.47	1.5	51.07	37.5	2
18	34	58	Male	170	72	24.9134948	2.86	48.12	55.79	7.5	49.52	36.58	3
19	58	70	Female	152	50	21.6412742	4.68	36.16	53.7	10.4	48.26	34.69	2
20	12	65	Female	158	59	23.634033	2.64	38.07	56.45	8.52	68.81	59.79	2
21	50	62	Female	151	55	24.121749	4.15	59.37	75.27	11.44	59.69	45.91	3
22	54	65	Female	153	58	24.7767952	5.13	43.15	50.68	8.29	81.37	68.58	2
23	34	69	Male	175	75	24.4897959	5.13	49.88	57.77	11.36	42.08	24.9	4
24	56	66	Female	159	61	24.1287924	6.58	44.61	60.91	10.71	44.95	30.63	3
25	62	56	Female	158	59	23.634033	3.44	26.05	33.96	6.96	52.85	38.04	2
26	44	63	Female	158	59	23.634033	4.33	27.93	41.52	5.65	73.98	63.44	3
27	72	61	Male	165	68	24.9770432	5.92	29.83	46.96	11.13	56.51	43.42	3
28	8	67	Female	155	59	24.5577523	3.61	36.25	59.4	10.35	71.21	60.85	3
29	50	62	Male	170	71	24.567474	5.17	42.88	60.64	8.07	61.77	48.19	2
30	22	57	Female	164	68	25.2825699	2.63	45.85	62.98	10.78	68.4	56.92	4
31	4	69	Female	160	63	24.609375	5.4	49.8	67.36	14.35	51.68	37.91	2
32	94	66	Male	170	70	24.2214533	4.74	52.15	70.18	1.5	42.46	25.11	3
33	50	67	Female	164	68	25.2825699	4.82	28.81	45.71	1.5	30.6	19.31	2
34	70	63	Female	160	61	23.828125	4.86	28.12	41.98	9.82	55.09	42.21	3
35	36	74	Female	154	58	24.4560634	4.52	58.98	65.14	1.5	53.31	40.24	3
36	36	75	Male	175	79	25.7959184	6.03	36.79	55.81	9.4	45.59	32.16	3
37	38	62	Female	160	61	23.828125	3.66	26.62	39.33	10.09	64.23	51.53	3
38	90	58	Female	159	60	23.7332384	3.34	42.06	58.52	10.31	69.68	59.98	3
39	50	67	Female	151	55	24.121749	4.88	36.96	55.88	12.03	36.72	20.81	4
40	44	64	Female	155	58	24.1415193	5.51	64.05	70.34	16.1	38.37	22.12	2
41	30	70	Male	173	77	25.7275552	4.31	63.72	77.94	10.46	81.04	66.93	3
42	40	58	Female	151	55	24.121749	4.18	28.14	45.46	8.91	53.15	39.02	2
43	12	69	Female	158	55	22.0317257	4.5	49.78	65.93	9.68	60.31	46.5	2
44	32	59	Female	166	68	24.6770213	3.38	42.63	50.11	10.66	56.83	43.42	2
45	74	68	Male	172	75	25.3515414	4.68	47.21	63.89	4.75	44.37	28.1	3
46	40	61	Female	164	65	24.1671624	3.43	37.76	56	12.54	65.43	53.99	2
47	24	59	Female	165	65	23.8751148	3.33	33.45	52.39	3.47	38.99	22.63	3
48	22	59	Male	165	65	23.8751148	3.44	46.85	63.36	2.07	68.12	54.28	2
49	52	59	Female	162	65	24.7675659	5.83	51.16	69.96	6.31	58.84	44.54	4
50	48	61	Female	160	61	23.828125	5.04	47.33	64.56	4.23	62.04	48.7	3
mean		64.44		160.32	63.2	24.5262107	4.3574	44.2208	59.5784	7.9676	57.4212	44.1108	2.8
std		4.63534249		7.15944132	6.67233093	1.11489671	0.98438369	12.3374266	13.0003261	3.84074397	12.4625632	13.671485	0.69282032

Patients number	VAS Rest											
	POD 6 H	POD 12 H	POD 24 H	POD 48 H	POD 72 H	POD 2W	POD 3 M	POD 6 M	POD 1 Y	POD 2 Y	PRE	POD 6 H	POD 12 H
1	3	3	3	2	2	2	1	1	1	1	6	3	3
2	3	2	2	2	2	2	1	1	1	1	4	2	2
3	2	2	1	1	1	1	0	0	0	0	6	2	2
4	2	2	1	1	1	0	0	0	0	0	5	4	3
5	2	2	2	2	2	2	1	1	0	0	5	4	4
6	2	1	1	1	1	0	0	0	0	0	3	5	5
7	2	2	2	2	2	1	1	0	0	0	4	4	4
8	2	2	2	2	1	1	0	0	0	0	4	2	2
9	2	2	2	2	2	1	1	0	0	0	4	4	4
10	3	3	2	2	2	2	1	1	1	1	4	3	3
11	2	1	1	1	0	0	0	0	0	0	5	3	3
12	2	1	1	0	0	0	0	0	0	0	4	3	3
13	2	2	2	2	2	1	1	1	0	0	4	5	4
14	2	2	2	2	2	1	0	0	0	0	4	3	3
15	4	3	3	2	3	2	1	1	1	1	6	5	5
16	4	4	3	2	2	2	1	1	1	1	4	4	4
17	2	2	2	2	2	1	0	0	0	0	5	3	2
18	2	2	2	1	1	1	0	0	0	0	4	3	3
19	2	2	1	1	1	0	0	0	0	0	6	4	3
20	2	2	2	1	1	1	0	0	0	0	4	4	4
21	2	2	2	2	2	2	1	1	0	0	6	3	3
22	3	3	2	2	2	2	1	1	1	1	4	4	3
23	2	2	1	1	1	0	0	0	0	0	4	4	4
24	3	2	2	2	2	2	1	1	1	1	4	3	3
25	2	1	1	1	1	0	0	0	0	0	5	2	2
26	2	1	1	0	0	0	0	0	0	0	4	3	3
27	2	1	1	1	1	0	0	0	0	0	3	3	3
28	2	1	1	1	0	0	0	0	0	0	4	2	2
29	2	2	2	2	2	1	1	0	0	0	5	3	3
30	2	2	2	2	1	1	0	0	0	0	6	4	3
31	3	2	2	2	2	2	1	1	1	1	5	3	3
32	2	2	2	2	1	1	0	0	0	0	5	3	3
33	2	2	1	1	1	0	0	0	0	0	8	3	3
34	2	2	2	1	1	1	0	0	0	0	5	3	3
35	2	2	2	2	1	1	0	0	0	0	5	3	3
36	2	2	2	2	2	2	1	1	1	0	5	3	3
37	2	1	1	1	0	0	0	0	0	0	5	2	2
38	2	2	2	2	2	1	1	0	0	0	3	2	2
39	3	2	2	2	2	2	1	1	1	1	5	3	3
40	2	2	2	1	1	1	0	0	0	0	4	4	3
41	2	1	1	1	1	0	0	0	0	0	3	2	2
42	2	2	2	1	1	1	0	0	0	0	4	3	2
43	2	2	1	1	1	0	0	0	0	0	5	4	4
44	3	3	2	2	2	2	1	1	1	1	6	4	4
45	4	3	3	3	2	2	1	1	1	1	4	5	4
46	2	2	2	2	1	1	0	0	0	0	6	4	4
47	2	1	1	1	1	0	0	0	0	0	4	3	3
48	2	2	1	1	1	0	0	0	0	0	3	3	3
49	2	2	2	2	2	1	1	0	0	0	2	3	2
50	2	2	2	2	2	2	1	1	1	0	6	3	3
mean	2.28	1.96	1.74	1.54	1.38	0.98	0.42	0.32	0.26	0.22	4.58	3.28	3.08
std	0.56709788	0.63118935	0.59363288	0.60695964	0.68963759	0.78714675	0.49355851	0.46647615	0.43863424	0.4142463	1.07870292	0.8255907	0.77045441

Patients number	VAS Active								Opioid consumption				
	POD 24 H	POD 48 H	POD 72 H	POD 2W	POD 3 M	POD 6 M	POD 1 Y	POD 2 Y	POD 1 D	POD 3 D	PRE	POD 1 D	POD 2 D
1	3	3	3	2	2	1	1	1	0	0	90	110	110
2	2	2	1	1	1	0	0	0	0	0	70	90	90
3	2	2	1	0	1	0	0	0	0	0	70	90	90
4	3	3	3	2	2	2	1	1	5	10	90	110	120
5	3	3	3	3	2	2	1	1	10	20	100	110	120
6	4	4	4	3	3	2	2	2	20	40	120	120	130
7	4	3	3	3	3	2	2	2	12.5	25	120	120	120
8	2	2	2	1	1	0	0	0	0	0	70	90	100
9	4	3	3	3	3	2	2	2	10	20	120	110	120
10	3	3	2	2	2	1	1	1	0	0	90	100	110
11	2	2	2	2	1	1	1	0	0	0	80	100	110
12	2	2	2	2	1	1	0	0	0	0	80	100	100
13	4	4	4	3	3	2	2	2	10	20	120	120	130
14	3	3	3	2	2	1	1	1	0	0	90	110	110
15	4	4	4	4	3	2	2	2	20	40	120	120	130
16	4	3	3	3	3	2	2	2	12.5	30	120	120	120
17	2	2	2	2	1	1	0	0	0	0	80	90	100
18	3	2	2	2	2	1	1	1	0	0	90	100	110
19	3	3	3	2	2	2	1	1	5	10	90	110	120
20	4	3	3	3	3	2	2	2	5	10	120	120	125
21	3	2	2	2	2	1	1	0	0	0	90	100	110
22	3	3	3	2	2	2	1	1	7.5	20	90	110	120
23	4	3	3	3	3	2	2	1	15	30	120	110	120
24	2	2	2	2	1	1	0	0	0	0	80	100	100
25	2	2	1	0	0	0	0	0	0	0	70	90	90
26	3	2	2	2	2	1	1	0	0	0	90	100	110
27	3	3	3	2	2	2	1	1	5	10	90	110	120
28	2	2	2	1	1	0	0	0	0	0	70	90	100
29	3	2	2	2	1	1	1	0	0	0	80	100	110
30	3	3	3	3	2	2	1	1	5	10	90	110	120
31	3	3	3	2	2	1	1	1	0	0	90	110	110
32	3	3	3	2	2	1	1	1	0	0	90	110	110
33	3	3	3	2	2	1	1	1	0	0	90	110	120
34	3	2	2	2	1	1	1	0	5	10	80	100	110
35	2	2	2	2	1	1	0	0	0	0	80	100	100
36	3	3	2	2	2	1	1	1	0	0	90	100	110
37	2	2	2	2	1	0	0	0	0	0	80	90	100
38	2	2	2	2	1	0	0	0	0	0	80	90	100
39	3	3	3	2	2	1	1	1	0	0	90	110	110
40	3	3	3	2	2	2	1	1	7.5	15	90	110	120
41	2	2	2	1	1	0	0	0	0	0	80	90	100
42	2	2	2	2	1	1	0	0	0	0	80	100	100
43	4	3	3	3	3	2	2	1	10	20	120	110	120
44	3	3	3	3	2	2	2	1	5	10	110	110	120
45	4	4	3	3	3	2	2	2	10	20	120	120	125
46	3	3	3	3	2	2	2	1	5	10	110	110	120
47	2	2	2	2	1	1	0	0	0	0	80	100	100
48	3	3	2	2	2	1	1	1	0	0	90	100	110
49	2	2	2	2	1	1	0	0	0	0	80	90	100
50	2	2	2	2	1	1	1	0	0	0	80	100	110
mean	2.86	2.64	2.5	2.14	1.8	1.22	0.94	0.74	3.7	7.6	92.2	104.4	111.2
std	0.72138755	0.62481997	0.7	0.74859869	0.77459667	0.70114193	0.73239334	0.71582121	5.41387107	11.1013513	16.1604455	9.62496753	10.4670913

Patients number	ROM						EES				[illegible]（day）	HSS	
	POD 3 D	POD 2W	POD 3 M	POD 6 M	POD 1 Y	POD 2 Y	POD 0 D	POD 1 D	POD 2 D	POD 3M		PRE	POD 6M
1	120	120	120	120	125	125	6	4	3	3	2	47	87
2	100	100	110	110	110	110	3	2	2	2	3	56	96
3	100	100	100	110	110	110	2	2	2	2	3	56	96
4	120	125	125	125	130	130	6	5	4	3	3	38	86
5	120	130	125	130	130	130	7	5	4	3	3	38	84
6	130	130	130	130	130	130	7	6	6	4	2	31	78
7	125	130	130	130	130	130	7	6	5	3	4	35	83
8	100	110	110	110	120	120	3	3	2	2	3	54	95
9	125	130	130	130	130	130	7	6	4	3	3	35	83
10	120	120	120	120	120	120	5	4	3	3	2	48	89
11	110	120	120	120	120	120	5	4	3	3	3	52	92
12	100	120	120	120	120	120	5	4	3	2	2	53	92
13	130	130	130	130	130	130	7	6	5	3	3	31	80
14	120	120	120	120	120	120	6	4	3	3	3	48	88
15	130	130	130	130	130	130	8	8	6	4	3	31	78
16	130	130	130	130	130	130	7	6	5	3	2	34	83
17	100	110	120	120	120	120	4	3	2	2	3	54	94
18	110	120	120	120	120	120	5	4	3	3	2	49	90
19	120	120	120	125	130	130	6	5	4	3	3	38	86
20	130	130	130	130	130	130	7	6	5	3	4	34	80
21	110	120	120	120	120	120	5	4	3	3	2	50	90
22	120	120	120	125	130	130	6	5	4	3	3	38	87
23	125	130	130	130	130	130	7	6	4	3	2	35	84
24	110	120	120	120	120	120	5	4	3	3	3	52	92
25	100	100	100	110	110	110	2	2	2	1	2	56	96
26	110	120	120	120	120	120	5	4	3	3	3	50	90
27	120	120	120	125	125	125	6	5	4	3	2	42	87
28	100	110	110	110	110	110	3	2	2	2	3	56	96
29	110	120	120	120	120	120	5	4	3	3	3	50	90
30	120	125	125	130	130	130	6	5	4	3	2	38	85
31	120	120	120	120	125	125	6	4	3	3	3	47	87
32	120	120	120	120	125	125	6	4	3	3	3	48	87
33	120	120	120	125	125	125	6	5	4	3	3	45	87
34	110	120	120	120	120	120	5	4	3	3	3	51	90
35	110	120	120	120	120	120	5	4	3	3	3	52	92
36	110	120	120	120	120	120	5	4	3	3	4	49	89
37	100	110	120	120	120	120	4	3	2	2	2	54	94
38	100	110	120	120	120	120	3	3	2	2	3	54	94
39	120	120	120	125	125	125	6	5	4	3	3	46	87
40	120	125	125	130	130	130	6	5	4	3	3	38	85
41	100	110	120	120	120	120	3	3	2	2	2	54	95
42	100	120	120	120	120	120	4	3	3	2	3	53	93
43	125	130	130	130	130	130	7	6	4	3	3	37	84
44	120	130	130	130	130	130	7	5	4	3	2	38	84
45	130	130	130	130	130	130	7	6	5	3	2	34	80
46	120	130	125	130	130	130	7	5	4	3	3	38	84
47	100	120	120	120	120	120	5	4	3	2	3	53	92
48	110	120	120	120	120	120	5	4	3	3	3	49	89
49	100	110	120	120	120	120	4	3	2	2	3	54	94
50	110	120	120	120	120	120	5	4	3	3	2	52	91
mean	114.2	120.3	121.3	122.6	123.4	123.4	5.38	4.36	3.4	2.76	2.74	45.5	88.3
std	10.3130985	8.0876449	6.69402719	6.01996678	5.95315043	5.95315043	1.44069428	1.26111062	1.03923048	0.54990908	0.55892754	8.07031598	4.92036584

Patients number			PCS			MCS		
	POD 1 Y	POD 2 Y	POD 6M	POD 1 Y	POD 2 Y	POD 6M	POD 1 Y	POD 2 Y
1	89	89	21	22	22	25	26	27
2	98	98	28	29	29	28	30	30
3	98	98	28	29	29	28	30	30
4	88	88	20	21	21	22	24	24
5	86	88	19	20	20	21	23	24
6	80	85	16	17	19	18	20	20
7	85	85	18	19	20	20	20	22
8	97	97	27	28	28	28	30	30
9	85	88	18	19	20	20	20	24
10	91	93	21	22	22	25	26	27
11	94	93	24	25	25	26	28	28
12	94	94	24	26	26	27	28	28
13	82	85	16	17	19	18	20	20
14	90	93	21	22	22	25	26	27
15	80	85	16	17	19	18	20	20
16	85	85	18	19	19	20	20	22
17	96	95	26	26	28	28	30	30
18	92	93	23	24	24	25	27	27
19	88	90	20	21	21	23	25	24
20	82	85	18	19	19	19	20	22
21	92	93	23	24	24	25	27	28
22	89	90	20	21	22	23	25	24
23	86	88	18	20	20	21	23	24
24	94	93	24	25	26	27	28	28
25	98	98	29	30	30	28	30	30
26	92	93	23	24	24	26	28	28
27	89	89	20	21	22	24	26	26
28	98	98	27	28	28	28	30	30
29	92	93	23	24	24	26	28	28
30	87	88	19	20	20	22	24	24
31	89	89	20	22	22	24	26	27
32	89	89	21	22	22	25	26	27
33	89	89	20	22	22	24	26	26
34	92	93	23	24	24	26	28	28
35	94	93	24	25	26	27	28	28
36	91	93	21	22	24	25	26	27
37	96	96	26	26	28	28	30	30
38	96	96	26	27	28	28	30	30
39	89	89	20	22	22	24	26	26
40	87	88	19	20	20	22	24	24
41	97	97	26	27	28	28	30	30
42	95	95	25	26	28	28	29	30
43	86	88	18	20	20	21	23	24
44	86	88	18	20	20	21	23	24
45	82	85	18	19	19	18	20	22
46	86	88	19	20	20	21	23	24
47	94	93	24	26	26	27	28	28
48	91	93	22	23	24	25	26	27
49	96	95	25	26	28	28	29	30
50	93	93	24	25	25	26	28	28
mean	90.3	91.2	21.74	22.86	23.36	24.2	25.82	26.32
std	4.92036584	3.96988665	3.41648943	3.32270974	3.34520552	3.20624391	3.30266559	2.94916937
